# Acceptability of an mHealth App That Provides Harm Reduction Services Among People Who Inject Drugs: Survey Study

**DOI:** 10.2196/25428

**Published:** 2021-07-14

**Authors:** Tyler Shelby, Xin Zhou, Douglas Barber, Frederick Altice

**Affiliations:** 1 Yale University School of Medicine New Haven, CT United States; 2 Department of Epidemiology of Microbial Diseases Yale University School of Public Health New Haven, CT United States; 3 Section of Infectious Diseases Yale University School of Medicine New Haven, CT United States; 4 Centre of Excellence on Research on AIDS University of Malaya Kuala Lumpur Malaysia

**Keywords:** people who inject drugs, mHealth, patient preferences, syringe services programs, service access, mobile phone

## Abstract

**Background:**

Harm reduction services reduce the negative consequences of drug injection and are often embedded within syringe service programs (SSPs). However, people who inject drugs (PWID) suboptimally engage with such services because of stigma, fear, transportation restrictions, and limited hours of operation. Mobile health (mHealth) apps may provide an opportunity to overcome these barriers and extend the reach of SSPs beyond that of the traditional brick-and-mortar models.

**Objective:**

This study aims to assess the prevalence of smartphone ownership, the level of comfort in providing the personal information required to use mHealth apps, and interest in using an mHealth app to access harm reduction services among PWID to guide the development of an app.

**Methods:**

We administered a survey to 115 PWID who were enrolled via respondent-driven sampling from July 2018 to July 2019. We examined the extent to which PWID had access to smartphones; were comfortable in providing personal information such as name, email, and address; and expressed interest in various app-based services. We measured participant characteristics (demographics, health status, and behaviors) and used binary logistic and Poisson regressions to identify independent correlates of mHealth-related variables. The primary regression outcomes included summary scores for *access*, *comfort*, and *interest*. The secondary outcomes included binary survey responses for individual comfort or interest components.

**Results:**

Most participants were White (74/105, 70.5%), male (78/115, 67.8%), and middle-aged (mean=41.7 years), and 67.9% (74/109) owned a smartphone. Participants reported high levels of comfort in providing personal information to use an mHealth app, including name (96/109, 88.1%), phone number (92/109, 84.4%), email (85/109, 77.9%), physical address (85/109, 77.9%), and linkage to medical records (72/109, 66.1%). Participants also reported strong interest in app-based services, including medication or sterile syringe delivery (100/110, 90.9%), lab or appointment scheduling (90/110, 81.8%), medication reminders (77/110, 70%), educational material (65/110, 59.1%), and group communication forums (64/110, 58.2%). Most participants were comfortable with the idea of home delivery of syringes (93/109, 85.3%). Homeless participants had lower access to smartphones (adjusted odds ratio [AOR] 0.15, 95% CI 0.05-0.46; *P*=.001), but no other participant characteristics were associated with primary outcomes. Among secondary outcomes, recent SSP use was positively associated with comfort with the home delivery of syringes (AOR 3.29, 95% CI 1.04-10.3 *P*=.04), and being older than 50 years was associated with an increased interest in educational materials (AOR 4.64, 95% CI 1.31-16.5; *P*=.02) and group communication forums (AOR 3.69, 95% CI 1.10-12.4; *P*=.04).

**Conclusions:**

Our findings suggest that aside from those experiencing homelessness or unstable housing, PWID broadly have access to smartphones, are comfortable with sharing personal information, and express interest in a wide array of services within an app. Given the suboptimal access to and use of SSPs among PWID, an mHealth app has a high potential to address the harm reduction needs of this vulnerable population.

## Introduction

### Background

The current opioid epidemic has resulted in many adverse outcomes, including increases in hospitalizations attributed to opioid injection and increases in infections of hepatitis C virus (HCV) and HIV, endocarditis, soft tissue infections, and overdose death [1-4]. Reductions in HIV incidence have stalled as a consequence of the volatile opioid epidemic, and numerous outbreaks of HIV and HCV have been reported in both urban and rural settings [4,5]. Although the increasing rates of infection among people who inject drugs (PWID) are staggering, approximately 130 individuals die from overdose each day in the United States [6]. This ongoing crisis calls for further implementation and development of novel methods to reduce risk among PWID and increase the delivery of harm reduction services.

To reduce the incidence of such negative health outcomes, syringe service programs (SSPs) offer services including syringe exchange; the provision of clean injection supplies, fentanyl test kits, and bandages; naloxone (Narcan) training and kit provision; and linkage to internal or external programs for housing support, mental health counseling, primary care, and addiction treatment. Through such a wide range of services, SSPs have demonstrated efficacy in reducing rates of HIV transmission [7] and syringe sharing [8] and increasing HIV treatment and prevention cascades [9-11]. However, SSPs’ uptake and coverage are far from meeting recommended targets and provide consistent services to only a quarter of PWID [12]. Reasons for low uptake include (1) long geographical distance to SSPs [13-15], especially in rural areas where opioid use is rising substantially [15]; (2) limited SSP hours of operation [16]; (3) lack of public transportation [17]; (4) the limited power of SSP workforces [18]; and (5) perceived stigma among PWID and fear of arrest and police interference [8,19-22]. These reasons may largely be attributed to the operational style of current SSPs, that is, services and supplies are either provided at central SSP locations or delivered through mobile vans at selected sites during scheduled hours [23]. Although home delivery and contact-free SSPs have been suggested as alternatives to overcome these barriers, they remain underexplored [24,25].

With regard to improving the accessibility and coverage of harm reduction services, a mobile health (mHealth) app has great potential to provide PWID with better access to health care and harm reduction services while also protecting their privacy and offering them a better sense of control of their environment. PWID increasingly have access to smartphones [26], and mHealth interventions have demonstrated significant potential to positively impact a variety of health conditions, including obesity prevention [27], physical activity and healthy eating promotion [28,29], cerebral stroke detection and management [30], and diabetes management [31,32], among others. However, the variety and quantity of mHealth apps for HIV care and prevention are still very limited. Only 18 were available in 2018 [33], and these apps mainly focus only on 2 functionalities: (1) self-management and self-monitoring tools for increasing opioid users’ adherence to medication [33-36] and (2) tools that can improve linkage and retention in HIV care among people with HIV [37,38]. None of the existing HIV-related apps have combined essential functionalities that are desired among individuals who seek HIV care and prevention, including scheduling appointments; viewing medication logs, lab reports, and current pharmacy information; tracking nutrition and fitness; and exchanging social support with other users, along with links to local resources and health information and support for self-managing stress and depression [33,37].

### Study Objectives

Given the potential of mHealth apps to increase effectiveness and access to HIV care and harm reduction, we sought to better understand its feasibility and acceptance among a group of individuals who have substantial risk for HIV, that is, PWID. In addition, we sought to understand whether any participant characteristics were associated with feasibility and acceptability or with particular services that could be offered in an mHealth platform. Herein, we present and discuss our findings, focusing on the following three dimensions of feasibility and acceptability: access, comfort, and interest.

## Methods

### Recruitment

From June 2018 to June 2019, 115 PWID were screened for eligibility in this study at the New Haven Syringe Service Program (NHSSP). The eligibility criteria were as follows: (1) being 18 years or older; (2) being able to understand, speak, and read English or Spanish; (3) self-reporting as an active injection drug user within the past 60 days; and (4) having at least one injection partner. Respondent-driven sampling (RDS) was used for recruitment, and the original *seeds* were recruited from clientele who use the NHSSP. *Seeds* were recruited using flyers distributed at the central and mobile distribution locations of the NHSSP.

Once enrolled, *seeds* were asked to complete a cross-sectional egocentric survey in-person at the NHSSP. An iPad (Apple Inc) was used to display survey questions which were hosted on Qualtrics (SAP) to the participants, and the study staff conducting the interview selected the respondents’ answers. Upon completion of the survey, each *seed* was compensated US $20 for their time. Following the interview, RDS was used to recruit subsequent waves of participants from within the *seed’s* current injection network (ie, individuals who had injected drugs with the participant within the past 2 months) using the same aforementioned eligibility criteria. Participants were allowed to recruit injection partners that were either currently engaged with or not engaged with the SSP. This allowed our sample to expand beyond the limits of the SSP clientele. Each *seed* was given US $10 for each successful network referral, and each referee was given US $20 upon completion of the survey. Once these network referees completed the survey, they were also asked to recruit their own injection network partners following the same RDS protocol. This resulted in subsequent waves of recruitment and expansion of the participant sample beyond the network of the original *seed*. All study protocols were approved by the Yale Human Subjections Committee, and a certificate of confidentiality was obtained from the National Institutes of Health to further protect participant information.

### Measures

#### Access to Mobile Devices

We dichotomously assessed *access* to mobile devices by asking participants if they had access to a cellphone without an internet connection, a cellphone with an internet connection (smartphone), a tablet, or a computer. The main item of interest in this survey was a smartphone, which would be required for the hypothetical service app.

#### Comfort Levels

We assessed *comfort* levels associated with using an app and providing personal information by asking participants whether they would be willing or unwilling to provide various personal information to use the app, including their name, phone number, email, address, alternative address, and linkage to medical records. Participants also indicated their comfort level with home delivery of syringes, rated on a 5-point Likert scale, with 1 being *very comfortable* and 5 being *very uncomfortable*; a grouped outcome variable for this item was created in which *very comfortable* and *comfortable* responses were coded as 1 and all other responses were coded as 0. A summary score for *comfort* was created by adding up participant responses for each item, thus transforming it to a 0-7 scale, with higher scores indicating a higher level of comfort.

#### Interest

We assessed interest in potential mHealth app services by asking participants to rate the usefulness of various services, including delivery of medications or syringes, scheduling appointments with providers and labs, setting medication reminders, accessing educational material about health and safe injection practices, and accessing group communication forums with peers. Participants indicated their level of perceived usefulness on a 5-point Likert scale, with 1 being *extremely useful* and 5 being *not at all useful*; a grouped variable for each service was created in which *extremely useful* and *very useful* responses were coded as 1 and all other responses were coded as 0. In addition, 3 survey items focused on the delivery of various services (pre-exposure prophylaxis [PrEP] medication, sterile syringes, and medication for opioid use disorder), and 2 survey items focused on scheduling appointments with providers or labs were combined into 2 grouped outcomes focused on either delivery or scheduling. We created a summary score for *interest* by adding up the binary scores described above, thus creating a 0-5 scale, with higher scores indicating a higher level of interest in comprehensive services.

#### Participant Characteristics

We collected self-reported data on demographics, health information, and behavioral history. We measured gender identity, race and ethnicity, levels of education, and housing status (stably housed vs homeless or unstably housed) categorically and later dichotomized education at the level of high school completion. We collected age data continuously and later categorized them into the following age groups: 18-34 years, 35-49 years, and ≥50 years. We measured perceived financial stability on a 10-item Likert scale, with 1 being *always worried about food, housing, and income* and 10 being *never worried about food, housing, and income*. We later dichotomized the responses for perceived financial stability at the median level. We measured the history of incarceration, syringe sharing with current injection partners, carrying of Narcan during injection, and recent engagement with the SSP in the previous 60 days dichotomously. We similarly collected dichotomous health information, including HCV and HIV status and overdose history.

### Statistical Analysis

Our primary outcomes included binary *access* to smartphones and summary scores for *comfort* and *interest*. We coded the primary *access* outcome as 1 if the participant owned a smartphone and 0 otherwise, using multivariable logistic regression when evaluating correlates. We used multivariable Poisson regression to evaluate correlations with summary scores for *comfort* and *interest*. In addition, to further explore any possible association between individuals’ characteristics and their *comfort* and *interest*, we used logistic regression to evaluate correlations with each individual survey item within the *comfort* and *interest* subsections. This allowed us to see which services on the mHealth app would appeal more to certain subsets of the PWID population. As we did not correct for multiple outcomes, these secondary analyses were considered hypothesis-generating. In all models, covariates with *P*<*.*20 in bivariate analyses were included in the final models for each outcome. We used complete case analysis, excluding observations with missing covariate or outcome data from the bivariate or multivariable models. All statistical analyses were conducted using Stata 16 (StataCorp).

## Results

### Participant Characteristics

Table 1 contains the original Likert scores for the items of *interest* and *comfort* with home syringe delivery. Roughly 5.2% (6/115) of participants did not answer any questions about mHealth access, comfort (6/115, 5.2%), and interest (5/115, 4.3%), and were therefore excluded from the regression models. In general, participants were in their early 40s, primarily White, male, and had completed high school; most described themselves as homeless or unstably housed, with a minority reporting being infected with HIV or HCV. Most participants reported a history of sharing syringes with their current injection partners and overdose. In addition, just above half (64/115, 55.7%) of the participants reported using the SSP within the past 60 days, and less than half (49/115, 42.6%) of the participants reported carrying Narcan when they inject (Table 2).

**Table 1:** Frequencies and percentages of Likert responses for component interests and comfort engaging with home syringe delivery (n=110).

Interests^a^	Extremely useful, n (%)	Very useful, n (%)	Moderately useful, n (%)	Slightly useful, n (%)	Not at all useful, n (%)
Delivery of PrEP^b^ services (n=108)	26 (24.1)	32 (29.6)	35 (32.4)	5 (4.6)	10 (9.3)
Delivery of syringes	47 (42.7)	45 (40.9)	13 (11.8)	1 (0.9)	4 (3.6)
Delivery of medications for opioid use disorder	57 (51.8)	32 (29.1)	11 (10)	1 (0.9)	9 (8.2)
Linking to electronic medical record to schedule appointments with clinical provider	43 (39.1)	41 (37.3)	20 (18.2)	5 (4.6)	1 (0.9)
Being scheduled for laboratory testing (eg, HIV, hepatitis C virus, and sexually transmitted infections; n=109)	44 (40.4)	41 (37.6)	17 (15.6)	5 (4.6)	2 (1.8)
Reminders to take medication	43 (39.1)	34 (30.9)	17 (15.5)	11 (10)	5 (4.6)
Receive educational materials	33 (30)	32 (29.1)	29 (26.4)	7 (6.4)	9 (8.2)
Group communication forums	34 (30.9)	30 (27.3)	29 (26.4)	8 (7.27)	9 (8.2)
Comfort with home syringe delivery^c^ (n=109)	45 (41.3)	48 (44)	2 (1.8)	8 (7.3)	6 (5.5)

^a^Unless specified as comfort rather than interest.

^b^PrEP: pre-exposure prophylaxis.

^c^This Likert scale included *very comfortable*, *comfortable*, *unsure*, *uncomfortable*, and *very uncomfortable*.

**Table 2:** Characteristics and survey responses of study participants (N=115).

Participant characteristics					Values
**Demographic characteristics**					
	**Age (years)**				
		Value, mean (SD)		41.7 (10.6)	
		**Age group, n (%)**			
			18-34	37 (32.2)	
			35-49	48 (41.7)	
			≥50	30 (26.1)	
	**Sex, n (%)**				
		Male		78 (67.8)	
	Hispanic ethnicity, n (%)			27 (23.5)	
	**Race (n=105), n (%)**				
		White		74 (70.5)	
		Black or African American		17 (16.2)	
		Other		14 (13.3)	
	Financial stability score, median (Q1, Q3)			3 (1, 5)	
	Completed high school, n (%)			87 (75.7)	
	Currently homeless or unstably housed, n (%)			72 (62.6)	
	Ever incarcerated, n (%)			96 (83.5)	
**Health status and behaviors**					
	Years of injecting, mean (SD)			14 (11.9)	
	Self-reported HIV positivity (n=110), n (%)			9 (8.2)	
	Self-reported hepatitis C virus positivity, n (%)			35 (30.4)	
	Syringe sharing with current injection partners, n (%)			101 (87.8)	
	Carry Narcan while injecting, n (%)			49 (42.6)	
	Recent syringe service program use, n (%)			64 (55.7)	
	Overdose, ever, n (%)			70 (60.9)	
**Mobile health opportunities (n=109), n (%)**					
	**Access to**				
		Cellphone without internet		12 (11)	
		Smartphone		74 (67.9)	
		Tablet		2 (1.8)	
		Computer		6 (5.5)	
		None		22 (20)	
	**Comfort with sharing personal information**				
		Name		96 (88.1)	
		Personal phone number		92 (84.4)	
		Personal email		85 (78)	
		Home address		85 (78)	
		Alternative address		62 (56.9)	
		Medical records		72 (66.1)	
	Comfort with home delivery of syringes^a^			93 (85.3)	
	**Component interests for an app^a^ (n=110)**				
		Delivery (PrEP^b^, syringes, and medications for opioid use disorder)		100 (90.9)	
		Clinical scheduling (provider and lab)		90 (81.8)	
		Medication reminders		77 (70)	
		Health education		65 (59.1)	
		Group communication forums		64 (58.2)	
**Mobile health** **summary scores for access, comfort, and interest (n=109)**					
	Access to smartphone, n (%)			74 (67.9)	
	Comfort (0-7), mean (SD)			5.4 (1.8)	
	Interest (0-5; n=110), mean (SD)			3.6 (1.5)	

^a^See Table 1 for full Likert score responses. The proportion of participants who perceived home delivery of syringes as *comfortable* or *very comfortable* and the proportion of those who perceived services as *very* or *extremely useful* are shown here.

^b^PrEP: pre-exposure prophylaxis.

### Access

Most participants owned a smartphone (74/109, 67.9%). As shown in Table 3, age, perceived financial stability, and homelessness or unstable housing reached *P*<.20 in the bivariate regression and were included in the multivariable model. In the final model, those who were homeless or unstably housed had significantly lower odds of smartphone ownership than those who were stably housed (adjusted odds ratio [AOR] 0.15, 95% CI 0.05-0.46; *P*=.001). Bivariate analyses are presented in Multimedia Appendix 1.

**Table 3:** Multivariable logistic and Poisson models for primary outcomes^a,b^.

Covariates			Primary Outcomes												
			Access to smartphone					Comfort providing personal identifiers and engaging with personalized services					Interest in comprehensive mobile health services		
			AOR^c^ (95% CI)		*P* value		Coefficient (95% CI)			*P* value		Coefficient (95% CI)			*P* value
**Age (years)**															
	18-34	Ref^d^		Ref		—^e^			—		Ref			Ref	
	35-49	2.78 (0.99 to 7.82)		.05		—			—		0.05 (−0.19 to 0.30)			.68	
	≥50	1.04 (0.32 to 3.43)		.95		—			—		0.21 (−0.07 to 0.48)			.14	
Financial stability >3			1.74 (0.66 to 4.59)		.27		—			—		—			—
Completed high school			—		—		0.13 (−0.07 to 0.33)			.19		—			—
*Currently homeless or unstably housed* ^f^			*0.15 (0.05 to 0.46)*		*.001*		—			—		—			—
HIV positivity			—		—		—			—		0.15 (−0.21 to 0.51)			.42

^a^Logistic regression used for *access* primary outcome.

^b^Poisson regression used for *comfort* and *interest* primary outcomes.

^c^AOR: adjusted odds ratio.

^d^Ref: reference group.

^e^Not available. The covariate was not included in the final model because it did not meet the bivariate threshold of *P*<.20.

^f^Italicized text denotes significance (*P*<.05).

### Comfort

Overall, most participants reported being comfortable with providing their names, phone numbers, email, address, access to medical records, and an alternative address such as a post office box on the mHealth app. In addition, a majority of participants reported being comfortable with using the home delivery of syringes on the app. Bivariate Poisson regressions showed that only education was associated with summary *comfort* scores with *P<.*02, although it was not statistically significant. No additional covariates were included in the final Poisson model.

### Interest

Most participants showed interest in the proposed app features. Specifically, delivery services were perceived as at least *very useful* by most participants, as were scheduling services, setting medication reminders, accessing educational materials related to health and safe injection practices, and accessing group communication and support forums. Table 1 highlights the additional percentage of respondents that indicated only a moderate or slight interest in each service. Age and HIV status were associated with overall *interest* summary scores in bivariate Poisson regression with *P<.*20 but were not significant in the final multivariable model.

### Secondary Analyses: Individual Comfort and Interest Item Analysis

Tables 4 and 5 present the multivariable findings from the secondary hypothesis-generating regression analyses. The bivariate results are presented in Multimedia Appendices 2 and 3. A few of these analyses revealed significant correlations. The middle-aged group (35-49 years) had higher odds of reporting comfort with providing their email than those who were younger (18-34 years; AOR 4.06, 95% CI 1.14-14.51; *P*=.03). In addition, those who completed high school had higher odds of reporting comfort with providing their medical records on the app than those with less education (AOR 2.87, 95% CI 1.12-7.39; *P*=.03). Those who engaged with the SSP at least once in the past 60 days had higher odds of reporting comfort with the idea of a doorstep-styled delivery of syringes (AOR 3.29, 95% CI 1.04-10.34; *P*=.04). Older PWID (≥50 years) had higher odds of reporting interest in having educational materials (AOR 4.64, 95% CI 1.31-16.46; *P*=.02) and communication forums on the app (AOR 3.69, 95% CI 1.10-12.44; *P*=.04). Those who had been previously incarcerated had lower odds of reporting interest in educational materials on the app (AOR 0.22, 95% CI 0.05-0.87; *P*=.03).

**Table 4:** Secondary multivariable logistic regression models evaluating individual comfort items.

Covariates		Individual comfort items															
		Name		Email			Phone number			Address			Medical records			Home syringe delivery	
		AOR^a^ (95% CI)	*P* value	AOR (95% CI)	*P* value	AOR (95% CI)		*P* value	AOR (95% CI)		*P* value	AOR (95% CI)		*P* value	AOR (95% CI)		*P* value
**Age (years)**																	
	18-34	Ref^b^	Ref	Ref	Ref	—^c^		—	Ref		Ref	—		—	—		—
	*35-49* ^d^	0.51 (0.09-2.92)	.45	*4.06 (1.14-14.51)*	*.03*	—		—	2.26 (0.75-6.78)		.15	—		—	—		—
	≥50	0.49 (0.05-5.23)	.56	0.77 (0.25-2.39)	.65	—		—	1.22 (0.39-3.81)		.74	—		—	—		—
Female		—	—	—	—	—		—	2.05 (0.69-6.13)		.20	—		—	—		—
Hispanic ethnicity		—	—	0.36 (0.11-1.12)	.08	—		—	—		—	—		—	—		—
*Completed high school*		1.74 (0.48-6.26)	.40	—	—	—		—	—		—	*2.87 (1.12-7.39)*		*.03*	—		—
Homeless or unstably housed		0.20 (0.39-1.00)	.05	—	—	—		—	—		—	0.52 (0.22-1.27)		.15	—		—
Ever been incarcerated		—	—	2.50 (0.78-8.10)	.13	—		—	—		—	—		—	—		—
Years of injecting		0.96 (0.90-1.03)	.29	—	—	—		—	—		—	0.98 (0.95-1.02)		.37	—		—
*Recent SSP use* ^e^		—	—	—	—	1.79 (0.61-5.24)		.29	—		—	—		—	*3.29 (1.04-10.34)*		*.04*
Carry Narcan		—	—	—	—	1.96 (0.62-6.15)		.25	—		—	—		—	—		—
Syringe sharing		—	—	—	—	—		—	—		—	2.21 (0.67-7.26)		.19	2.69 (0.70-10.37)		.15

^a^AOR: adjusted odds ratio.

^b^Ref: reference group.

^c^Not available. The covariate was not included in the final model because it did not meet the bivariate threshold of *P*<.20.

^d^Italicized text denotes significance (*P*<.05).

^e^SSP: syringe service program.

**Table 5:** Secondary multivariable logistic regression models evaluating individual interest items.

Covariates			Individual interest items																		
			Delivery				Scheduling				Reminders				Educational material				Communication forums		
			AOR^a^ (95% CI)		*P* value		AOR (95% CI)		*P* value		AOR (95% CI)		*P* value		AOR (95% CI)		*P* value		AOR (95% CI)		*P* value
**Age (years)**																					
	18-34	—^b^		—		—		—		Ref^c^		Ref		Ref		Ref		Ref		Ref	
	35-49	—		—		—		—		1.79 (0.65-4.91)		.26		0.97 (0.36-2.59)		.95		1.23 (0.46-3.29)		.68	
	≥*50*^d^	—		—		—		—		5.05 (0.95-26.72)		.06		*4.64 (1.31-16.46)*		*.02*		*3.69 (1.10-12.44)*		*.04*	
**Race**																					
	White	Ref		Ref		—		—		Ref		Ref		—		—		Ref		Ref	
	Black or African American	0.37 (0.07-1.96)		.24		—		—		1.59 (0.38-6.65)		.52		—		—		1.31 (0.37-4.63)		.67	
	Other	0.28 (0.04-1.91)		.20		—		—		1.15 (0.34-3.97)		.82		—		—		1.13 (0.32-4.02)		.85	
Female			2.90 (0.32-26.86)		.35		—		—		—		—		—		—		—		—
Hispanic ethnicity			—		—		0.44 (0.14-1.33)		.15		—		—		2.17 (0.69-6.75)		.18		2.01 (0.57-7.02)		.28
Completed high school			—		—		1.88 (0.62-5.70)		.27		—		—		—		—		—		—
*Ever been incarcerated*			—		—		—		—		—		—		*0.22 (0.05-0.87)*		*.03*		—		—
HIV positivity			—		—		—		—		—		—		3.52 (0.36-34.62)		.28		—		—
HCV^e^positivity			—		—		—		—		—		—		—		—		0.46 (0.19-1.14)		.09
Years of injecting			0.97 (0.92-1.03)		.34		—		—		1.00 (0.95-1.05)		.99		—		—		—		—
Carry Narcan			3.29 (0.61-17.73)		.17		—		—		—		—		0.57 (0.24-1.38)		.21		—		—

^a^AOR: adjusted odds ratio.

^b^Not available. The covariate was not included in the final model because it did not meet the bivariate threshold of *P*<.20.

^c^Ref: reference group.

^d^Italicized text denotes significance (*P*<.05).

^e^HCV: hepatitis C virus.

## Discussion

### Principal Findings

Our results demonstrate a high prevalence of smartphone ownership and diffuse comfort and interest associated with mHealth services among the PWID included in this study. The results of our primary analyses were largely null aside from finding that those that were homeless or unstably housed had lower odds of smartphone ownership. In combination with the high rates of reported comfort and interest, this suggests that such an app-based approach would be acceptable for a wide range of PWID. The hypothetical services included in our survey would each offer unique methods of increasing harm reduction service uptake in the PWID community by overcoming various barriers to SSP engagement and warrant further research and development in this area.

Few significant correlations were found in our primary regression analyses, indicating that the acceptability of an mHealth app is diffusely similar across subsets of PWID. Although most participants reported low financial stability, most owned smartphones and also reported being comfortable with providing their personal information such as name, phone number, email, and address to be able to use an mHealth app. The smartphone ownership level among participants was high but lower than the 81% reported nationally in the United States [39]. Smartphone ownership was lower among those experiencing homelessness or unstable housing, indicating that reaching this group with an mHealth app could be challenging. The comfort associated with providing linkage to medical records was slightly lower than the other comfort survey items, indicating that integrating electronic health records in the app could be a barrier for those who have privacy concerns. This may more commonly include those with lower levels of education, as suggested by our secondary analyses. Nonetheless, two-thirds of the participants were comfortable with providing access to medical records and could benefit from linking harm reduction to health care services. Most participants showed a strong desire for a contact-free format to obtain syringes and medications via the app. This service could address current barriers (eg, limited hours and personnel and stigmatization) associated with the current in-person, face-to-face format of most SSPs. Overall, the mostly null findings from our primary analyses indicate a widespread acceptability of an mHealth app designed for PWID.

Our secondary hypothesis-generating analyses revealed significant associations between age and interest in educational materials and group discussion forums and comfort with providing access to personal email. We also found education to be associated with comfort with providing linkage to medical records and a history of incarceration to be associated with interest in educational materials. Although only hypothesis-generating, these secondary findings indicate that allowing customization in an app is crucial for a positive user experience, especially for those with particular interests or privacy concerns. Of particular importance is the secondary finding that recent SSP use increased the odds of comfort with the home delivery of syringes. Aligned with the theory of diffusion by Roger [40,41], this finding indicates that PWID who are currently engaged with SSPs are likely to be the key influencers of the diffusion of an mHealth app and may drive the initial adoption of an mHealth app because of their established trust and familiarity with harm reduction providers and services. The subsequent diffusion of the app to PWID not currently engaged with SSPs may rely on early adopters to encourage their social network contacts to adopt the app.

The variety and quantity of mHealth apps designed for PWID and HIV or HCV care and prevention are very limited [33,42]. Most current apps serve as self-monitoring tools to increase adherence to medication among opioid users or people with HIV [33-36]. To our knowledge, there are no mHealth apps that use a patient-to-provider model offering broad and comprehensive services for PWID, which would greatly advance the mHealth field given the high levels of medical and psychiatric comorbidity in this population [43]. Our findings indicate that a multifunctional mHealth app that can deliver medication reminders, educational materials, and communication forums is highly desired by PWID, the latter especially for older PWID. This app may help reduce the risk of adverse health outcomes and build upon the core harm reduction services such as medication (eg, antiretroviral therapy, PrEP, HCV antivirals, and buprenorphine) and syringe provision [33]. Providing medication reminders for PWID, especially tailored messages, on an app may improve their self-management and medication adherence, which is particularly critical for PWID taking PrEP or antiretroviral therapy [44,45]. In addition, educational materials would also offer PWID information about how to reduce their risks while injecting and educate about overdose prevention and naloxone use. As drug injection is an illicit and often taboo activity, PWID must carefully navigate their environments while seeking information about or assistance with safe injection practices. This is particularly the case for those who have recently transitioned to drug injection, a growing subgroup of PWID that experience a particularly high risk of adverse outcomes, especially overdose, because of gaps in health knowledge, inexperience, and risk behaviors [46]. Our study extends this literature by showing that such education is also important for older PWID, and an mHealth app may be an effective, highly preferred platform for delivering educational material. By providing such educational material within an app, PWID can deploy safe injection practices using instructions within their reach. Finally, our study shows that communication forums may also be favored by older PWID. This demonstrates the older PWID’s desire for web-based communication and support. Such communication forums with peers may offer PWID a means to exchange information on safe injection practices and lessons learned and also provide encouragement and emotional support in a safe, monitored environment.

### Limitations

Despite the many new findings, this study has several limitations. This study included a relatively small sample size, which may have resulted in type II errors, and the fact that all initial *seed* PWID were enrolled from the SSP clientele may have resulted in sampling bias. Previous evaluation of RDS methods suggest that 4-6 recruitment waves are needed to overcome sampling bias with regard to race, sex, and drug use status [47], but the mean number of recruitment waves per *seed* in our study was 1.59. However, the most productive network in this study consisted of 12 waves and produced 56.5% (65/115) of the study participants, and a total of 83.5% (96/115) of our sample stemmed from seeds with at least three recruitment waves, suggesting that we were able to overcome some, but not all, of the sampling bias.

### Strengths of This Work

A strength of our study was our ability to recruit and interview PWID who do not currently engage with the local SSP; 46.1% (53/115) of the participants were not currently engaged with the NHSSP. These participants represent a large and hidden population of PWID that is not reached by the current brick-and-mortar SSP models. Their responses regarding their preferences and barriers to using SSPs give us crucial insight into what improvements are needed to increase the accessibility of SSPs to better address the health needs of this population, including through mHealth delivery strategies. Furthermore, this study provided insight into a broad range of relevant issues regarding mHealth feasibility and interest, including access to required devices, comfort levels, and interest.

### Conclusions

In conclusion, our findings suggest high acceptability of an mHealth app among PWID with little differences between subgroups of PWID, suggesting that an app would not only be applicable to a niche of PWID. The barriers to traditional SSP engagement, including awareness, access, and stigma, may be circumvented with such a novel intervention, and offering alternative solutions via a mobile platform could greatly increase the reach of SSPs and reduce PWIDs’ risk of harm significantly. Further research is warranted on this topic, and in-depth interviews and focus-group discussions should be used to provide more detailed information about how such an app could best serve the target audience.

## Appendix

Multimedia Appendix 1 Bivariate logistic and Poisson models for primary outcomes.

Multimedia Appendix 2 Secondary bivariate logistic regression models evaluating individual comfort items.

Multimedia Appendix 3 Secondary bivariate logistic regression models evaluating individual interest items.

## Abbreviations

AOR: adjusted odds ratio
HCV: hepatitis C virus
mHealth: mobile health
NHSSP: New Haven Syringe Service Program
PrEP: pre-exposure prophylaxis
PWID: people who inject drugs
RDS: respondent-driven sampling
SSP: syringe service program
