# Supplementary material for: Acceptability of an mHealth App That Provides Harm Reduction Services Among People Who Inject Drugs: Survey Study
Source: J Med Internet Res. 2021 Jul 14;23(7):e25428. doi: 10.2196/25428 (PMC8319773; doi:10.2196/25428)
Supplement: Multimedia Appendix 1 [file jmir_v23i7e25428_app1.docx]

Supplementary Table 1: Bivariate logistic^a^ and Poisson^b^ models for primary outcomes.

| Covariates | | Primary Outcomes | | | | | |
| --- | --- | --- | --- | --- | --- | --- | --- |
|  | | Access to smartphone | | Comfort providing personal identifiers and engaging with personalized services | | Interest in comprehensive mHealth^c^ services | |
|  | | AOR^d^  (95% CI) | P | Coefficient  (95% CI) | P | Coefficient  (95% CI) | P |
|  | |  |  |  |  |  |  |
| Age | | | | | | | |
|  | 18-34 | Ref^e^ | Ref | Ref | Ref | Ref | Ref |
|  | 35-49 | 2.51  (0.96-6.55) | .06 | 0.06  (−0.13-0.25) | .55 | 0.08  (-0.16-0.32) | .51 |
|  | ≥50 | 1.75  (0.62-4.95) | .29 | −0.04  (−0.26-0.17) | .70 | 0.25  (−0.004-0.51) | .05 |
| Race | | | | | | | |
|  | White | Ref | Ref | Ref | Ref | Ref | Ref |
|  | Black or African American | 0.84  (0.28-2.56) | .76 | −0.15  (−0.25-0.22) | .90 | 0.08  (−0.20-0.35) | .58 |
|  | Other | 1.03  (0.29-3.71) | .96 | 0.03  (−0.23-0.28) | .83 | -0.02  (−0.33-0.29) | .92 |
| Female | | 1.28  (0.53-3.07) | .59 | 0.03  (−0.14-0.20) | .71 | 0.13  (−0.08-0.33) | .24 |
| Hispanic ethnicity | | 1.19  (0.44-3.21) | .73 | −0.05  (−0.25-0.15) | .63 | 0.06  (−0.17-0.29) | .63 |
| Financial stability >3 | | 2.07  (0.89-4.82) | .09 | −0.02  (−0.19-0.14) | .79 | −0.002  (−0.20-0.20) | .98 |
| Completed high school | | 1.45  (0.58-3.63) | .43 | 0.13  (−0.07-0.33) | .19 | 0.003  (−0.23-0.23) | .98 |
| Currently homeless or unstably housed | | *0.16*  *(0.06-0.45)* | *.001* | −0.09  (−0.26-0.07) | .26 | −0.04  (−0.25-0.16) | .67 |
| Ever been incarcerated | | 0.55  (0.17-1.82) | .33 | 0.02  (−0.20-0.24) | .86 | −0.08  (−0.34-0.18) | .57 |
| HIV+ | | 1.57  (0.30-8.22) | .59 | 0.08  (−0.22-0.38) | .62 | 0.24  (−0.10-0.58) | .17 |
| HCV^d^+ | | 0.58  (0.25-1.38) | .22 | -0.01  (−0.19-0.16) | .87 | −0.05  (−0.26-0.17) | .68 |
| Years of injecting | | 1.01  (0.97-1.04) | .72 | −0.003  (−0.01-0.004) | .42 | 0.003  (−0.01-0.01) | .52 |
| Recent SSP Use | | 0.78  (0.35-1.77) | .56 | 0.07  (−0.09-0.24) | .38 | −0.002  (−0.20-0.20) | 0.98 |
| Carry Narcan | | 1.60  (0.70-3.65) | .26 | 0.03  (−0.13-0.20) | .68 | −0.03  (−0.23-0.16) | .73 |
| Syringe sharing | | 1.71  (0.54-5.36) | .36 | 0.05  (−0.20-0.30) | .70 | 0.06  (−0.25-0.36) | .72 |
| Overdose history | | 0.77  (0.33-1.77) | .53 | 0.01  (−0.16-0.18) | .90 | −0.04  (−0.24-0.16) | .69 |

*Italicized text* denotes significance (P<.05)

^a^Logistic regression used for *access* primary outcome.

^b^Poisson regression used for *comfort* and *interest* primary outcomes.

^c^mHealth: mobile health.

^d^AOR: adjusted odds ratio.

^e^Ref denotes the reference group.
