# Supplementary material for: Acceptability of an mHealth App That Provides Harm Reduction Services Among People Who Inject Drugs: Survey Study
Source: J Med Internet Res. 2021 Jul 14;23(7):e25428. doi: 10.2196/25428 (PMC8319773; doi:10.2196/25428)
Supplement: Multimedia Appendix 2 [file jmir_v23i7e25428_app2.docx]

Supplementary Table 2: Secondary bivariate logistic regression models evaluating individual comfort items

| Covariates | | Individual Comfort Items | | | | | | | | | | | |
| --- | --- | --- | --- | --- | --- | --- | --- | --- | --- | --- | --- | --- | --- |
|  | | Name | | Email | | Phone number | | Address | | Medical records | | Home  Syringe delivery | |
|  | | AOR^a^  (95% CI) | P | AOR  (95% CI) | P | AOR  (95% CI) | P | AOR  (95% CI) | P | AOR  (95% CI) | P | AOR (95% CI) | P |
| Age | | | | | | | | | | | | | |
|  | 18-34 | Ref^b^ | Ref | Ref | Ref | Ref | Ref | Ref | Ref | Ref | Ref | Ref | Ref |
|  | 35-49 | 0.42  (0.08-2.21) | .30 | 3.42  (1.04-11.18) | .04 | 0.96  (0.28-3.33) | .95 | 2.32  (0.78-6.92) | .13 | 0.90  (0.35-2.30) | .82 | 1.43  (0.42-4.89) | .57 |
|  | ≥50 | 0.30  (0.05-1.68) | .17 | 0.93  (0.31-2.72) | .89 | 0.83  (0.21-3.20) | .78 | 1.31  (0.42-4.04) | .64 | 0.91  (0.32-2.60) | .86 | 1.34  (0.34-5.30) | .68 |
| Race | | | | | | | | | | | | | |
|  | White | Ref | Ref | Ref | Ref | Ref | Ref | Ref | Ref | Ref | Ref | Ref | Ref |
|  | Black or African American | 0.60  (0.14-2.56) | .49 | 2.41  (0.50-11.60) | .27 | 1.25  (0.25-6.32) | .79 | 0.71  (0.22-2.32) | .57 | 1.02  (0.34-3.09) | .97 | 0.54  (0.15-2.00) | .36 |
|  | Other | 1.55  (0.18-13.55) | .69 | 0.72  (0.20-2.64) | .62 | 0.56  (0.13-2.38) | .43 | 1.63  (0.33-8.13) | .55 | 0.89  (0.26-3.01) | .85 | 0.92  (0.18-4.77) | .92 |
| Female | | 1.67  (0.43-6.49) | .46 | 0.73  (0.29-1.89) | .52 | 1.65  (0.50-5.49) | .41 | 2.07  (0.70-6.11) | .19 | 1.18  (0.50-2.79) | .70 | 1.05  (0.33-3.29) | .94 |
| Hispanic ethnicity | | 0.93  (0.24-3.70) | .92 | 0.46  (0.17-1.27) | .14 | 0.62  (0.20-1.99) | .43 | 0.81  (0.28-2.33) | .69 | 1.04  (0.40-2.70) | .94 | 1.26  (0.33-4.86) | .73 |
| Financial stability >3 | | 1.83  (0.53-6.34) | .34 | 0.70  (0.28-1.74) | .44 | 2.02  (0.66-6.19) | .22 | 0.87  (0.35-2.16) | .76 | 0.71  (0.32-1.58) | .40 | 1.31  (0.44-3.92) | .62 |
| Completed high school | | 2.23  (0.66-7.54) | .20 | 1.43  (0.52-3.96) | .49 | 1.96  (0.65-5.97) | .23 | 1.43  (0.52-3.96) | .49 | 3.04  (1.23-7.55) | .02 | 1.08  (0.31-3.67) | .91 |
| Currently homeless or unstably housed | | 0.24  (0.05-1.16) | .08 | 0.90  (0.35-2.29) | .83 | 1.09  (0.38-3.12) | .87 | 0.56  (0.21-1.49) | .25 | 0.53  (0.23-1.23) | .14 | 0.91  (0.30-2.71) | .86 |
| Ever been incarcerated | | 0.91  (0.18-4.50) | .91 | 2.77  (0.94-8.19) | .07 | 0.63  (0.13-3.05) | .57 | 1.46  (0.46-4.60) | .52 | 1.29  (0.46-3.67) | .63 | 0.30  (0.04-2.41) | .26 |
| HIV+ | | 0.91  (0.10-8.07) | .93 | 2.08  (0.24-17.84) | .50 | 1.30  (0.15-11.31) | .81 | 2.08  (0.24-17.84) | .50 | 1.65  (0.31-8.60) | .56 | O.^c^ | O. |
| HCV^d^+ | | 1.44  (0.37-5.63) | .60 | 0.62  (0.24-1.61) | .32 | 1.00  (0.32-3.10) | .99 | 0.79  (0.30-2.08) | .63 | 1.46  (0.59-3.59) | .41 | 0.90  (0.29-2.84) | .86 |
| Years of injecting | | 0.96  (0.92-1.00) | .07 | 1.00  (0.96-1.04) | .87 | 1.00  (0.96-1.04) | .92 | 0.99  (0.95-1.03) | .58 | 0.98  (0.95-1.01) | .19 | 0.98  (0.94-1.02) | .37 |
| Recent SSP Use | | 1.57  (0.49-5.01) | .45 | 0.88  (0.35-2.21) | .79 | 2.03  (0.71-5.81) | .19 | 1.69  (0.68-4.20) | .26 | 1.12 | .77 | 3.33  (1.07-10.37) | .04 |
| Carry Narcan | | 1.35  (0.41-4.44) | .62 | 0.77  (0.31-1.91) | .57 | 2.20  (0.72-6.75) | .17 | 1.19  (0.47-2.97) | .71 | 1.11  (0.50-2.47) | .80 | 1.06  (0.36-3.08) | .92 |
| Syringe sharing | | 1.27  (0.25-6.45) | .77 | 1.50  (0.43-5.29) | .53 | 0.89  (0.18-4.38) | .89 | 0.96  (0.25-3.76) | .96 | 2.17  (0.70-6.73) | .18 | 2.77  (0.75-10.23) | .13 |
| Overdose history | | 0.99  (0.30-3.28) | .99 | 0.95  (0.37-2.41) | .91 | 1.14  (0.40-3.27) | .81 | 0.95  (0.37-2.41) | .91 | 1.35  (0.60-3.03) | .47 | 1.29  (0.44-3.77) | .64 |

^a^AOR: adjusted odds ratio.

^b^Ref denotes the reference group.

^c^O. denotes that covariate was omitted due to perfect prediction.

^d^HCV: hepatitis C virus.
