# Supplementary material for: Acceptability of an mHealth App That Provides Harm Reduction Services Among People Who Inject Drugs: Survey Study
Source: J Med Internet Res. 2021 Jul 14;23(7):e25428. doi: 10.2196/25428 (PMC8319773; doi:10.2196/25428)
Supplement: Multimedia Appendix 3 [file jmir_v23i7e25428_app3.docx]

Supplementary Table 3: Secondary bivariate logistic regression models evaluating individual interest items

| Covariates | | Individual Interest Items | | | | | | | | | |
| --- | --- | --- | --- | --- | --- | --- | --- | --- | --- | --- | --- |
|  | | Delivery | | Scheduling | | Reminders | | Educational material | | Communication forums | |
|  | | AOR^a^  (95% CI) | P | AOR  (95% CI) | P | AOR  (95% CI) | P | AOR  (95% CI) | P | AOR  (95% CI) | P |
| Age | | | | | | | | | | | |
|  | 18-34 | Ref^b^ | Ref | Ref | Ref | Ref | Ref | Ref | Ref | Ref | Ref |
|  | 35-49 | 1.42  (0.27-7.50) | .68 | 1.50  (0.50-4.50) | .47 | 1.86  (0.74-4.68) | .19 | 1.04  (0.43-2.52) | .93 | 1.14  (0.47-2.75) | .78 |
|  | ≥50 | 0.60  (0.12-2.96) | .54 | 1.92  (0.51-7.20) | .33 | 4.93  (1.41-17.29) | .01 | 4.80  (1.48-15.54) | .01 | 3.14  (1.06-9.29) | .04 |
| Race | | | | | | | | | | | |
|  | White | Ref | Ref | Ref | Ref | Ref | Ref | Ref | Ref | Ref | Ref |
|  | Black or African American | 0.20  (0.04-0.89) | .04 | 1.88  (0.38-9.17) | .44 | 2.59  (0.68-9.89) | .16 | 1.07  (0.37-3.14) | .90 | 2.14  (0.68-6.72) | .19 |
|  | Other | 0.36  (0.06-2.21) | .27 | 1.50  (0.30-7.48) | .62 | 1.00  (0.30-3.31) | .99 | 0.75  (0.24-2.37) | .62 | 1.19  (0.37-3.79) | .77 |
| Female | | 4.64  (0.56-38.13) | .15 | 1.505  (0.50-4.52) | .47 | 1.69  (0.67-4.25) | .27 | 1.51  (0.65-3.47) | .34 | 1.59  (0.69-3.66) | .28 |
| Hispanic ethnicity | | 1.19  (0.24-6.03) | .83 | 0.35  (0.12-0.99) | .047 | 1.13  (0.42-3.04) | .80 | 2.08  (0.79-5.50) | .14 | 2.18  (0.83-5.76) | .12 |
| Financial stability >3 | | 0.46  (0.12-1.75) | .26 | 0.89  (0.34-2.37) | .82 | 0.85  (0.38-1.94) | .71 | 1.65  (0.76-3.61) | .21 | 0.95  (0.44-2.04) | .89 |
| Completed high school | | 1.36  (0.33-5.66) | .68 | 2.49  (0.89-6.96) | .08 | 0.98  (0.38-2.53) | .96 | 0.81  (0.33-1.97) | .64 | 0.62  (0.25-1.54) | .31 |
| Currently homeless or unstably housed | | 1.04  (0.28-3.93) | .95 | 0.81  (0.29-2.22) | .68 | 1.22  (0.53-2.80) | .64 | 0.66  (0.30-1.46) | .30 | 0.73  (0.33-1.60) | .43 |
| Ever been incarcerated | | 0.54  (0.06-4.57) | .57 | 0.88  (0.23-3.39) | .86 | 1.62  (0.56-4.62) | .37 | 0.36  (0.11-1.16) | .09 | 0.65  (0.22-1.88) | .43 |
| HIV+ | | 0.72  (0.08-6.49) | .77 | O.^c^ | O. | O. | O. | 5.35  (0.63-45.14) | .12 | 2.39  (0.46-12.43) | .30 |
| HCV^d^+ | | 1.80  (0.36-8.96) | .47 | 1.90  (0.58-6.20) | .29 | 0.80  (0.33-1.92) | .62 | 0.77  (0.34-1.75) | .53 | 0.48  (0.21-1.09) | .08 |
| Years of injecting | | 0.96  (0.91-1.01) | .10 | 1.01  (0.96-1.05) | .79 | 1.03  (0.99-1.07) | .13 | 1.01  (0.98-1.05) | .40 | 1.02  (0.98-1.05) | .31 |
| Recent SSP Use | | 1.33  (0.36-4.87) | .67 | 0.83  (0.31-2.23) | .72 | 1.32  (0.58-3.00) | .50 | 0.78  (0.36-1.68) | .52 | 0.99  (0.46-2.12) | .98 |
| Carry Narcan | | 3.55  (0.72-17.54) | .12 | 0.98  (0.37-2.59) | .96 | 0.95  (0.42-2.15) | .90 | 0.55  (0.25-1.18) | .12 | 0.80  (0.37-1.71) | .56 |
| Syringe sharing | | 1.83  (0.35-9.67) | .48 | 1.27  (0.32-5.04) | .74 | 0.60  (0.16-2.31) | .46 | 1.53  (0.50-4.70) | .46 | 1.46  (0.48-4.50) | .51 |
| Overdose history | | 1.09  (0.29-4.10) | .90 | 0.85  (0.31-2.33) | .75 | 1.29  (0.56-2.96) | .55 | 0.60  (0.27-1.33) | .21 | 0.78  (0.36-1.71) | .53 |

^a^AOR: adjusted odds ratio.

^b^Ref denotes the reference group.

^c^O. denotes that covariate was omitted due to perfect prediction.

^d^HCV: hepatitis C virus.
